# Bioinformatics for the synthetic biology of natural products: integrating across the Design–Build–Test cycle

**DOI:** 10.1039/c6np00018e

**Published:** 2016-05-17

**Authors:** Pablo Carbonell, Andrew Currin, Adrian J. Jervis, Nicholas J. W. Rattray, Neil Swainston, Cunyu Yan, Eriko Takano, Rainer Breitling

**Affiliations:** a Manchester Centre for Fine and Specialty Chemicals (SYNBIOCHEM) , Manchester Institute of Biotechnology , University of Manchester , Manchester M1 7DN , UK . Email: pablo.carbonell@manchester.ac.uk ; Email: eriko.takano@manchester.ac.uk

## Abstract

Covering: 2000 to 2016

Progress in synthetic biology is enabled by powerful bioinformatics tools allowing the integration of the design, build and test stages of the biological engineering cycle. In this review we illustrate how this integration can be achieved, with a particular focus on natural products discovery and production. Bioinformatics tools for the DESIGN and BUILD stages include tools for the selection, synthesis, assembly and optimization of parts (enzymes and regulatory elements), devices (pathways) and systems (chassis). TEST tools include those for screening, identification and quantification of metabolites for rapid prototyping. The main advantages and limitations of these tools as well as their interoperability capabilities are highlighted.

## Abstract 2

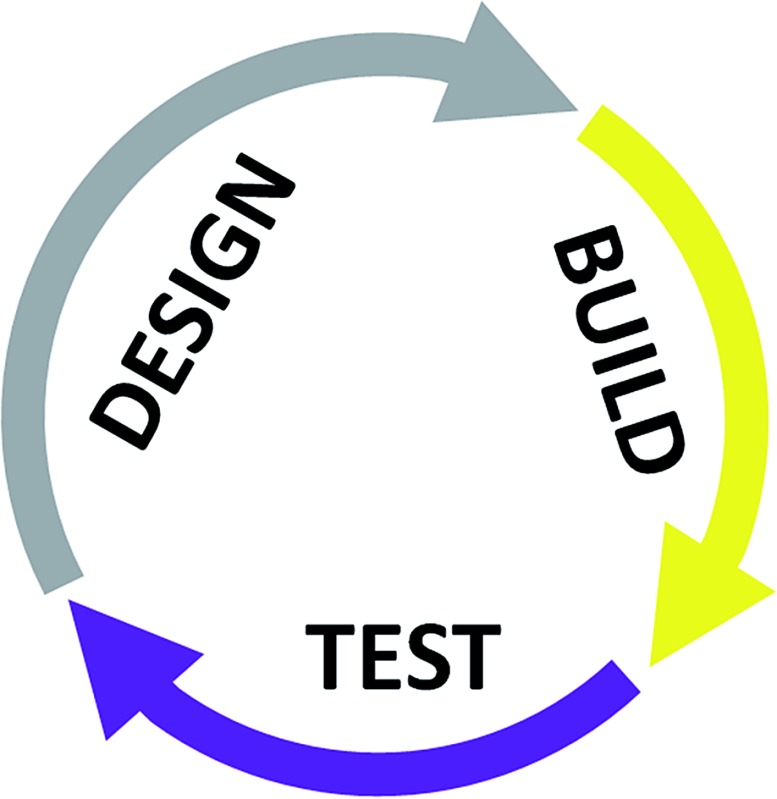
Bioinformatics tools facilitate and accelerate all steps along the Design–Build–Test cycle of synthetic biology, for the enhanced production of natural products in engineered microbes.

## 1 The DESIGN–BUILD–TEST cycle of synthetic biology

**Fig. 1:**
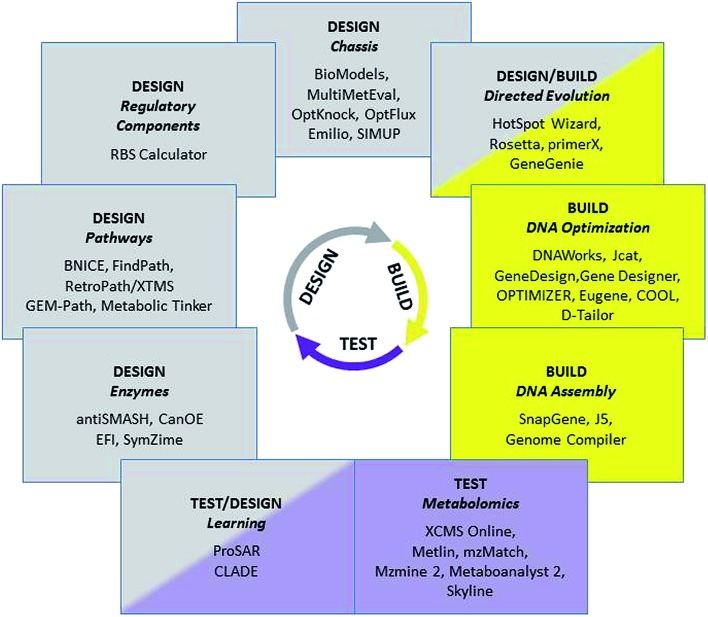
Selected bioinformatics tools and associated tasks for the Design/Build/Test cycle in synthetic biology.

More than 100 000 natural products, *i.e.* organic chemical compounds produced by living organisms, have been identified in the last 150 years, including highly diverse chemical classes such as polyketides, non-ribosomal peptides, phenylpropanoids, alkaloids or isoprenoids. These compounds are used in a wide range of interesting applications, ranging from pharmaceutical uses as drugs against many diseases to flavours and fragrances in food and personal care products. Their economic potential and the fact that they are originally synthesized by biological systems make natural products highly attractive targets for the advanced genetic engineering strategies of synthetic biology, with the aim of producing them more efficiently, in more amenable host species, from cheaper raw material, and potentially with the option of introducing added value and new functionalities by engineered modifications of the biosynthetic pathways. The necessary large-scale engineering of microbial production systems is only possible if it is supported by tailored computational tools at each of the stages of the engineering cycle (Fig. 1).

## 2 Computational tools for the DESIGN stage

Computational design tools are needed in order to identify the best combinations of enzymes, pathways, regulatory components, and chassis organisms leading to the efficient production of target natural products (see Table 1). This includes tools that mine databases for candidate parts, such as the antiSMASH software,^1^ which identifies and annotates biosynthetic gene clusters for natural products in sequenced microbial genomes. In a parallel strategy, tools for automated annotation and prediction of enzyme activity,^2^ such as CanOE Strategy^3^ and the Enzyme Function Initiative,^4^ are helping in the selection and design of best candidate enzymes for catalysing specific chemical reactions (including unnatural ones) to be added to engineered biosynthetic pathways.

**Table 1:** Overview of design tools for various levels of the synthetic biology hierarchy

	Enzymes	Pathways	Regulatory components	Chassis
Selection	*Mining*	*Ranking*	*Characterization*	*Genome-scale metabolic modeling*
	antiSMASH^1^	FindPath^12^	Registry of Standardised Biological Parts	BioModels^28^
		RetroPath^13^		MultiMetEval^30^
		GEM-Path^15^		
		Metabolic Tinker^11^		
Prediction	*Annotation*	*Search*	*Tuning*	*Optimization*
	antiSMASH^1^	BNICE^6^	RBS Calculator^19^	OptKnock^41^
	CanOE^3^	RouteSearch^8^		EMILiO^42^
	Enzyme Function Initiative^4^	PathPred^9^		SIMUP^43^
	SymZime^6^	RetroPath^13^		RobOKoD^44^
		GEM-Path^15^		

In an extreme variant of this approach, instead of modifying natural pathways, newly assembled enzymatic routes can be explored to produce a target natural product in a chassis organism.^5^ For this approach to be successful, tools are needed to systematically search for all possible pathways leading to a target compound, and to correctly prioritize them through a ranking algorithm based on predicted pathway's efficiency. BNICE and SimZyme^6^ are a collection of pathway design tools that apply a set of reaction rules to predict possible biosynthetic routes towards desired target compounds and then identify the candidate enzymes that might be coerced to catalyse the necessary reactions. This tool kit has been applied to predict possible biosynthetic pathways for target compounds starting from native metabolites; these are currently awaiting experimental verification. Other recent proposed tools for selecting the most promising enzymatic route towards a target include the Sympheny Biopathway Predictor^7^ developed by Genomatica; RouteSearch,^8^ based on atom mapping; and PathPred,^9^ based on the reaction patterns in the KEGG database. Work in this area is still in a very early stage: for instance, PathPred was used to look for alternative biosynthesis routes in the flavonoid pathways converting the plant pigment delphinidin into gentiodelphin.^9^ The system was able to predict new pathways in addition to known pathways, but most of them were found to be non-viable upon manual inspection because they required predicted reactions that are chemically infeasible. The main challenge of pathway prediction tools will be the automated prioritization of successful candidates from among the easily generated thousands of alternative pathways. Possible criteria for identifying a predicted pathway as likely to be efficient are diverse and several computational approaches to estimate pathway efficiency have been proposed.^10^ For instance, Metabolic Tinker^11^ prioritizes pathways based on thermodynamic feasibility; FindPath^12^ in addition considers pathway length and theoretical yield; RetroPath/XTMS^13,14^ scores enzyme performance based on predicted promiscuous activities and adds toxicity of intermediates to the ranking; and GEM-Path^15^ includes flux efficiency.

An important consideration when designing an engineered pathway is the selection of regulatory components. Pathway efficiency requires preventing flux imbalances, which would lead to the depletion of essential precursors or the accumulation of intermediates, which in turn could result in toxicity or feedback inhibition of the pathway. This can be achieved by the right selection of regulatory components including promoters and transcriptional terminators, and ribosome binding site, which control transcription and translation rates, respectively. The accurate prediction of promoter and terminator properties is not currently possible based on sequence data alone; instead libraries of promoters and terminators have been experimentally characterised and standardised to allow predictive selection. The necessary characterisation information is held in databases such as The Registry of Standardised Biological Parts (http://parts.igem.org/Main_Page), and in the primary literature.^16–18^ For the computational prediction of the properties of ribosome binding sites (RBS), the situation is slightly better, and there is a class of tools for engineering binding sites to achieve desired translation rates in prokaryotic hosts.^19–22^ Unlike other regulatory elements (promoters, terminators), RBS sites are strongly influenced by their flanking sequence, including the 5′ end of their cognate open reading frame (ORF) and, in operons, the 3′ terminus of the previous ORF.^23^ For this reason, the design of RBS sites should ideally be done simultaneously to ORF sequence optimization, but currently no tools are available to do this. So far, RBS prediction has been used successfully to debug aberrant RBS sites mid-ORF during sequence optimization,^24^ to design bespoke RBS,^25^ and to optimize RBS library design for the engineering of *E. coli* pathways to increase riboflavin levels^26^ and NADPH recycling.^27^

Generally, natural products of interest are not naturally produced by common industrial production microbes; instead their biosynthetic pathways need to be engineered for recombinant production in industry-compatible strains. A growing number of genome-scale metabolic models (GEMs), available at the BioModels repository,^28^ can assist in the selection of the optimal chassis strain for a specific natural product,^29,30^ and the subsequent optimization of chassis metabolism. Central to *in silico* approaches for chassis selection and optimization are constraint-based flux prediction approaches.^31^ The first requirement for the application of these approaches is the availability of comprehensive descriptions of the stoichiometry of all metabolic reactions in an organism, which can usually be inferred from genome annotations in combination with manual curation. The resulting models collate all known metabolic reactions – along with information on metabolic enzymes, transporters, and their encoding genes – in a principled format that is amenable to computational analysis.^32^ Such models have increased in scale, coverage and quality over the last 15 years and are now available for many organisms relevant to industrial biotechnology.^33,34^ Furthermore, protocols and automated computational pipelines for their construction have been published.^35–37^ To select suitable chassis strains for a particular natural product, the reactions of its biosynthetic pathway are added to the metabolic models of a collection of different potential hosts, and multi-objective optimization (*e.g.*, using the MultiMetEval software^30^) is applied to predict which strain can achieve the optimal balance between biomass production and the production of the desired chemical.

Even when a predicted optimal strain has been chosen for the engineered production of a natural product, additional rounds of strain optimization are usually required to reach industrially viable production levels. For this task, the same constraint-based metabolic models serve as the starting point. The basic premise of strain optimization is to amend host metabolism such that metabolic flux is increased towards the target molecule whilst maintaining cellular growth. This commonly involves the implementation of gene knockouts or over-expressions to channel metabolic flux as required.^38^ Constraint-based flux analysis can be used to predict which genes will be the most promising targets for this strategy, and a large number of tools have been developed to implement this approach.^39–43^

## 3 Computational tools for the BUILD stage

When introducing an engineered pathway into a new chassis strain, the applied genetic manipulations are no longer restricted to producing new combinations of selected pathway parts and regulatory elements. Instead, as DNA synthesis is increasingly affordable, it is possible to design the sequence of each individual part before combining them into optimized devices. At each step, multi-objective optimization is needed to ensure that synthetic genes express successfully in a given host, including organism-specific codon-optimization, alleviation of secondary mRNA structure, as well as removal of intrinsic regulation (transcriptional and translational), repeating sequences and homopolymeric tracts. Many pieces of freely available software can be combined in automated pipelines for sequence optimization, as shown in Fig. 1 (a detailed comparison of strengths and limitations of these tools has recently been provided by Gould *et al.* ^45^). Furthermore, many commercial gene synthesis vendors (including Gen9, GeneArt and GenScript) provide their own optimization algorithms for use prior to submitting orders, which allows further specific optimization for their synthesis methodology. All the available design tools allow codon optimization and the definition of specific base patterns such as restriction enzyme recognition sequences that should be avoided, but the rationale and algorithm for codon optimization and the degree of consideration of other criteria vary widely between programs.

Individual DNA parts will be assembled into larger constructs to produce biosynthetic pathways, and so the design of the part sequences should be compatible with the downstream assembly method (and ideally with multiple assembly methods to allow part sharing within the scientific community). For instance, removing all BsaI restriction sites makes parts compatible with GoldenGate assembly and variations,^46^ and the inclusion of unique ends facilitates seamless assembly methods such as Gibson and the Ligase Cycling Reaction.^47,48^ The process of defining the correct sequence for all parts and their intended combinations can be remarkably complex and error-prone, particularly when multiple or combinatorial assembly is to be performed. Design tools such as j5,^49^ SnapGene (; http://www.snapgene.com) and Genome Compiler (; http://www.genomecompiler.com/) have functions for schematic *in silico* pathway construction, which will automatically generate the required oligomer sequences including restriction sites, overhangs and linkers. Furthermore, “recipes” – instructions for the experimental order of assembly of parts *in vitro* – are produced by these tools in order to streamline the sequence ordering and experimental process. These design tools have functionality to design assemblies compatible with such protocols as GoldenGate,^46^ InFusion (; http://www.clontech.com), Gibson^47^ and Gateway cloning (; http://www.lifetechnologies.com), and functionality is constantly improving to support new assembly methods.

## 4 Computational tools at the interface of DESIGN and BUILD

A particular challenge for the engineering of natural products production involves those cases, where no suitable enzymes are available for a specific step within a pathway. This can be the case when the native enzyme that performs a particular transformation has not yet been identified, or when *de novo* pathways require chemical transformations not necessarily seen in nature (in the case of “unnatural” natural products). In these instances, directed evolution can be employed to engineer enzymes to improve their activity towards a predetermined reaction.^50^ This method involves a close interaction of designing and building (and ultimately testing), which require special computational tools that allow this direct connection between the stages of the engineering cycle. Directed evolution approaches generate variant libraries of a gene of interest, encoding an enzyme that is predicted to have at least some activity towards the desired reaction, and selects variants that exhibit an improved function. Iterative cycles of variation and selection can be employed until the desired fitness (*i.e.*, enzymatic activity) is reached. Traditionally, the necessary genetic diversity was achieved using random methods, primarily error-prone PCR^51–53^ or recombination,^54,55^ or site-directed mutagenesis (amongst others^56–58^). However, in the context of synthetic biology, gene synthesis^59^ approaches provide a means by which more rational strategies of protein engineering can be employed.

Sequence alignment tools like Clustal^60,61^ and MUSCLE,^62^ can analyse patterns of sequence diversity and conservation within classes of proteins, which can inform about the site and type of mutations that are most likely to lead to improved functionality. If a 3D structure of the protein that serves as the evolutionary starting point is known, then the HotSpot Wizard tool,^63^ which integrates functional, structural and evolutionary data, can be used to identify potential target residues.

Having decided upon the target residues and type of variants to create,^50^ the design tool GeneGenie^64^ can be used to guide the *de novo* synthesis of variant libraries. GeneGenie designs DNA sequences optimized for expression in a desired host, includes any sequences required for downstream cloning, and the mixed-base codon sequences. The resulting oligonucleotide sequences can then be synthesised and assembled using the SpeedyGenes method,^59^ which accommodates multiple and combinatorial variant sequences while at the same time implementing efficient enzymatic error correction, to create large but controlled libraries of variants, significantly reducing the “hands-on” time required for the experimental design.

## 5 Computational tools for the TEST stage

Following the construction of engineered microbial strains for natural product production, it is essential to characterize their phenotype in sufficient detail to provide informative feedback for the next iteration of the DESIGN stage. The major technological platform for this purpose are various molecular profiling methods, most importantly metabolomics. These methods not only help to characterize the production level of the target compound, but also allow a broad untargeted characterization of the metabolic state of the engineered microbe, which allows the detection of pathway bottlenecks,^65^ accumulation of unwanted intermediates, as well as unexpected pleiotropic consequences of the genetic manipulations. When focusing on quantitative profiling of changes in the composition of the growth medium (*i.e.*, the uptake and secretion rate of pathway products and precursors), metabolomics can also provide highly useful flux constraints to include in genome-scale metabolic models, which increases their predictive power for improved strain designs. The models can be further supplemented by constraints based on quantitative transcriptome profiles, which in microbes can serve as an informative proxy for enzyme activity levels and thus pathway flux.^66^

The development of computational tools for untargeted metabolomics is a mature area of research, and a large number of comprehensive software platforms have been made available in recent years (Table 2). However, all of the available tools still struggle with the increased throughput of the analytical instruments and the accelerated iterations of the synthetic biology engineering cycle. Particular challenges include the robust and reliable automated annotation of the detected metabolites and the direct integration of the results into improved models for the DESIGN stage.

**Table 2:** Open source software for untargeted/targeted MS analysis

Software name	Function, platform and output	Source
XCMS Online^67^	Framework for processing and visualization of LC-MS-based and single-spectrum mass spectral data – carries out nonlinear retention time alignment, feature detection, and feature matching. Open-source, hosted by the Bioconductor project (https://www.bioconductor.org/) that can be used in the R statistical package (; https://www.r-project.org/).	https://xcmsonline.scripps.edu/
Metlin	Metabolite ID platform hosted by the Scripps Research Institute and directly linked to XCMS Online. Contains data on over 240k metabolites that are linked to outside sources such as KEGG.	https://metlin.scripps.edu/index.php
mzMatch^68,69^	R and Java-based data processing platform that provides common tools for processing LC-MS data. Can extract, match, filter, and normalize peaks, and annotates them by matching to numerous *m*/*z* databases	http://mzmatch.sourceforge.net/
	Also available in a more user-friendly macro-enabled Excel format within the IDEOM platform. Can also integrate directly into XCMS.	
MZmine 2 (ref. 70)	Java-based pipeline from signal processing to statistical analysis and visualization. Utilizes the RANSAC algorithm for alignment and uses the PubChem and KEGG database (amongst others) for compound identification.	http://mzmine.github.io/
Metaboanalyst 3.0 (ref. 71 and 72)	Web-based server that supports LC-MS, GC-MS and NMR-based datasets. Contains modules for data processing, quality control and normalization, alongside a suite of univariate and multivariate chemometric analyses.	http://www.metaboanalyst.ca/
Mass Cascade^73^	The first published KNIME-based (https://www.knime.org/) metabolomics workflow that supports a broad range of flexible functionality. Can potentially link to XCMS, Matlab and R whilst at the same time having in-built nodes allowing the development of a fully customisable pipeline.	https://bitbucket.org/sbeisken/masscascadeknime/wiki/Home
		https://www.knime.org/

## 6 Tools at the interface of TEST and DESIGN

Computational tools to automate the feedback from the molecular characterization of engineered strains in the TEST stage to the improved engineering strategies of the DESIGN stage are one of the major remaining gaps in the computational synthetic biology toolbox. Few convincing examples exist at the moment, and even when computational tools are used, these tend to be bespoke scripts for a specific project, rather than generalized pipelines. Existing design tools still require a better coupling to screening and selection technologies. The development of high-throughput approaches to that end, including targeted biosensors^74^ or trackable gene traits,^75^ is necessary. Protocol languages for the automation of synthetic biology robotic platforms,^76^ such as the ones established by bio-foundries like Abolis, Zymergen, Ginkgo Bioworks, Amyris and SYNBIOCHEM, should facilitate the generalization of these pipelines. Moreover, integration of automated data analysis and machine-learning workflows into the protocols will ultimately provide the tools to seamlessly feed back from TEST into DESIGN.^77^ An area where rapid progress can be expected is the field of directed evolution for parts optimization; here, substantial datasets comprising quantitative sequence–activity information can often be obtained. In these cases, computational approaches, such as those implemented in the ProSAR software, can be adopted to infer predictive statistical models of sequence–activity relationships, to guide the next round of library design.^78–80^ Future improvements could include a more efficient mapping of an enzyme fitness landscape using machine learning algorithms, as has already been demonstrated in a related proof-of-concept for learning the sequence–activity relationship of DNA aptamers, using the Closed Loop Aptameric Directed Evolution (CLADE) approach.^81^

## 7 Conclusions

Efficient production of natural products in non-native chassis organisms is becoming more streamlined through the application of synthetic biology techniques. A growing range of computational tools is facilitating the synthetic biology engineering approach at each step of the process. However, the integration of DESIGN, BUILD and TEST tools is still one of the main challenges at present, and lack of interoperability between the bioinformatics tools is hindering a wider adoption of these tools by the community. Present requirements include a better standardisation to ensure interoperability between individual tools and seamless integration and traceability across the design/build/test stages. Several initiatives, like the NIST Synthetic Biology Standards Consortium, have recently been launched to address such standardisation issues.^82^ Of particular prominence for the establishment of computational standards is the Synthetic Biology Open Language (SBOL),^83^ an RDF-based standard for representing synthetic gene design that has been developed by an international consortium over recent years. The current release, SBOL v2.0,^84^ incorporates both structural and functional design features and integrates with systems biology modelling standards such as the Systems Biology Markup Language (SBML),^85^ providing a link between computational modelling (DESIGN) and wet-lab assembly (BUILD). SBOL is augmented with a visual representation, SBOL Visual^86^ which has the goal of standardising the visual representation of synthetic gene constructs, analogous to the standard representation of electronic circuits that enables electronic engineering. Moreover, optimization of the design process requires a better definition of constraints and objectives in a multiscale fashion. Such approaches would need to be matched by rapid prototyping systems for the BUILD stage exploring the design space efficiently. Similarly, autonomous and continuous learning from experimental test results needs to be enabled. Recently established bio-foundries, which are synthetic biology-based chemical manufacturers operating under tight and demanding constraints, serve as a critical testbed for computational tools at every step of the DESIGN–BUILD–TEST cycle and are key players in promoting the adoption of standard practices enabling software interoperability. It can be predicted that the experiences gained in these ambitious large-scale bio-engineering enterprises will rapidly diffuse to the wider synthetic biology community in the coming years.
